# Prevalence of hepatitis B and C viruses among migrant workers in Qatar

**DOI:** 10.1038/s41598-024-61725-9

**Published:** 2024-05-17

**Authors:** Gheyath K. Nasrallah, Hiam Chemaitelly, Ahmed I. A. Ismail, Parveen B. Nizamuddin, Duaa W. Al-Sadeq, Farah M. Shurrab, Fathima H. Amanullah, Tasneem H. Al-Hamad, Khadija N. Mohammad, Maryam A. Alabdulmalek, Reham A. Al Kahlout, Ibrahim Al-Shaar, Manal A. Elshaikh, Mazen N. Abouassali, Ibrahim W. Karimeh, Mutaz M. Ali, Houssein H. Ayoub, Sami Abdeen, Ashraf Abdelkarim, Faisal Daraan, Ahmed Ibrahim Hashim Elhaj Ismail, Nahid Mostafa, Mohamed Sahl, Jinan Suliman, Elias Tayar, Hasan Ali Kasem, Meynard J. A. Agsalog, Bassam K. Akkarathodiyil, Ayat A. Alkhalaf, Mohamed Morhaf M. H. Alakshar, Abdulsalam Ali A. H. Al-Qahtani, Monther H. A. Al-Shedifat, Anas Ansari, Ahmad Ali Ataalla, Sandeep Chougule, Abhilash K. K. V. Gopinathan, Feroz J. Poolakundan, Sanjay U. Ranbhise, Saed M. A. Saefan, Mohamed M. Thaivalappil, Abubacker S. Thoyalil, Inayath M. Umar, Einas Al Kuwari, Peter Coyle, Andrew Jeremijenko, Anvar Hassan Kaleeckal, Hanan F. Abdul Rahim, Hadi M. Yassine, Asmaa A. Al Thani, Odette Chaghoury, Mohamed Ghaith Al Kuwari, Elmoubasher Farag, Roberto Bertollini, Hamad Eid Al Romaihi, Abdullatif Al Khal, Mohamed H. Al-Thani, Laith J. Abu-Raddad

**Affiliations:** 1 Biomedical Research Center, Qatar University, Doha, Qatar; 2 Department of Biomedical Science, College of Health Sciences, QU Health, Qatar University, 2713 Doha, Qatar; 3 Infectious Disease Epidemiology Group, Weill Cornell Medicine-Qatar, Cornell University, P.O. Box 24144, Doha, Qatar; 4 World Health Organization Collaborating Centre for Disease Epidemiology Analytics on HIV/AIDS, Sexually Transmitted Infections, and Viral Hepatitis, Weill Cornell Medicine-Qatar, Cornell University, Qatar Foundation-Education City, Doha, Qatar; 5 Department of Population Health Sciences, Weill Cornell Medicine, Cornell University, New York, NY USA; 6 Laboratory Section, Medical Commission Department, Ministry of Public Health, Doha, Qatar; 7 Mathematics Program, Department of Mathematics and Statistics, College of Arts and Sciences, Qatar University, Doha, Qatar; 8 Hamad Medical Corporation, Doha, Qatar; 9 Ministry of Public Health, Doha, Qatar; 10 Qatar Red Crescent Society, Doha, Qatar; 11 Wellcome-Wolfson Institute for Experimental Medicine, Queens University, Belfast, UK; 12 Department of Public Health, College of Health Sciences, QU Health, Qatar University, Doha, Qatar; 13 Primary Health Care Corporation, Doha, Qatar; 14 College of Health and Life Sciences, Hamad Bin Khalifa University, Doha, Qatar

**Keywords:** Hepatitis, HBV, HCV, Prevalence, Infection, Workers, Cross-sectional, Qatar, Viral hepatitis, Epidemiology

## Abstract

Limited data exist on viral hepatitis among migrant populations. This study investigated the prevalence of current hepatitis B virus (HBV) infection and lifetime hepatitis C virus (HCV) infection among Qatar's migrant craft and manual workers (CMWs), constituting 60% of the country's population. Sera collected during a nationwide COVID-19 population-based cross-sectional survey on CMWs between July 26 and September 9, 2020, underwent testing for HBsAg and HCV antibodies. Reactive samples underwent confirmatory testing, and logistic regression analyses were employed to explore associations with HBV and HCV infections. Among 2528 specimens tested for HBV infection, 15 were reactive, with 8 subsequently confirmed positive. Three samples lacked sufficient sera for confirmatory testing but were included in the analysis through multiple imputations. Prevalence of current HBV infection was 0.4% (95% CI 0.2–0.7%). Educational attainment and occupation were significantly associated with current HBV infection. For HCV infection, out of 2607 specimens tested, 46 were reactive, and 23 were subsequently confirmed positive. Prevalence of lifetime HCV infection was 0.8% (95% CI 0.5–1.2%). Egyptians exhibited the highest prevalence at 6.5% (95% CI 3.1–13.1%), followed by Pakistanis at 3.1% (95% CI 1.1–8.0%). Nationality, geographic location, and occupation were significantly associated with lifetime HCV infection. HBV infection is relatively low among CMWs, while HCV infection falls within the intermediate range, both compared to global and regional levels.

## Introduction

Hepatitis B virus (HBV) and hepatitis C virus (HCV) infections present a global public health challenge ^1,2^, contributing substantially to morbidity and mortality, with the disease progression to fibrosis, cirrhosis, and liver cancer ^3–5^. Despite the potential for preventing severe outcomes associated with these infections, they persist as major global health concerns ^1–3^. The Global Burden of Disease Study has identified viral hepatitis as the seventh leading cause of death worldwide, with HBV and HCV making nearly equal contributions to this burden ^2^. The World Health Organization (WHO) formulated the Global Health Sector Strategy 2022–2030 with the aim of eliminating viral hepatitis as a public health concern by 2030 ^6^. Global targets were set, aiming for incidences of < 2 cases for HBV and < 5 cases per 100,000 for HCV, respectively ^6^.

The primary modes of transmission for HBV and HCV involve percutaneous or mucosal exposure to infected blood and bodily fluids, encompassing vertical transmission, parenteral routes (such as needlestick injuries and injection drug use), and sexual contact ^2,3,7^. The Middle East and North Africa (MENA) region show a considerable prevalence of HBV infection ^1^, while concurrently being the most affected global region by HCV infection ^8^.

Vaccination at birth effectively prevents HBV infection, yet vaccine coverage remains insufficient in low-income countries ^3^. The identification of current HBV infection relies on serological testing for the presence of the hepatitis B surface antigen (HBsAg) ^3^. According to the WHO, the global prevalence of HBsAg was estimated at 3.8% in 2019, indicating that 296 million individuals were enduring chronic HBV infections ^3,7^. The estimated annual number of new HBV infections in 2019 was 1.5 million ^7^.

While there is currently no vaccine available for HCV infection, the advent of the curative pan-genotypic direct-acting antivirals (DAAs) exhibiting a sustained virological response exceeding 95% across all HCV genotypes signifies a groundbreaking development in HCV treatment and control ^9,10^. The enhanced accessibility of DAAs, facilitated by generic formulations and substantially reduced prices in resource-limited countries ^11^, underscores the potential to eliminate HCV infection as a public health threat ^12,13^. HCV testing typically involves serological assays to identify HCV-specific antibodies, and individuals testing positive for antibodies undergo a molecular test to confirm the virus's presence and diagnose current infection ^14^. The WHO estimated the number of chronic HCV infections at 58 million in 2019, with an additional 1.5 million new infections during that year ^7^.

Against a backdrop of limited data on viral hepatitis among migrant populations worldwide, this study aimed to assess, to our knowledge for the first time, the prevalence of current HBV infection and lifetime HCV infection (indicating ever having been infected with HCV) within Qatar's craft and manual worker (CMW) population. Qatar, situated in the MENA region, has a young and diverse demographic, with only 9% aged 50 years or older and 89% comprising expatriates from over 150 countries ^15^. The CMW demographic, constituting approximately 60% of Qatar's total population, primarily consists of unmarried men aged 20–49 years, recruited for employment in infrastructure and development projects, including those associated with the World Cup 2022 ^16,17^. The overarching objective is to provide insights into HBV and HCV epidemiology, contributing data to national initiatives aimed at achieving global viral hepatitis elimination targets.

## Methods

### Study design and sampling

This study analyzed archived blood serum specimens obtained from a nationwide serological survey conducted between July 26, 2020, and September 09, 2020 ^16,18,19^. The primary objective was to determine the prevalence of antibodies against severe acute respiratory syndrome coronavirus 2 (SARS-CoV-2) infection among CMWs ^16^. CMWs comprised the most impacted demographic in Qatar's population during the first year of the coronavirus disease 2019 (COVID-19) pandemic ^15,20^.

The survey's sampling strategy was devised through an analysis of the registered users' database of the Qatar Red Crescent Society (QRCS), the primary healthcare provider for CMWs in the country ^16^. QRCS oversees four strategically located centers dedicated to addressing the healthcare needs of the CMW population nationwide ^16^. These centers, situated across Qatar, feature expansive catchment areas, operate extended hours, and offer services either free of charge or with substantial subsidies ^16^. To ensure sample representativeness, the probability distribution of CMWs based on age and nationality from the QRCS database was cross-referenced with that of expatriate residents from the Ministry of Interior database ^21^.

Since men comprise > 99% of CMWs ^22^, the sampling strategy did not explicitly consider sex. CMWs were recruited at QRCS centers using a systematic sampling approach, informed by the average daily attendance at each center ^16^. At each center, the recruitment process involved inviting every fourth attendee to participate in the study until the required sample size was achieved across all age and nationality strata. To address challenges in recruiting participants in smaller age-nationality strata, especially among younger individuals of certain nationalities, the recruitment criteria were adjusted towards the end of the study. In these instances, all attendees in these strata were invited to participate, rather than every fourth attendee.

### Sample collection and handling

Trained interviewers administered the written informed consent and the study instrument to participants, accommodating their language preferences among nine options: Arabic, Bengali, English, Hindi, Nepali, Sinhala, Tagalog, Tamil, and Urdu ^16^. The instrument, designed following WHO guidance for developing SARS-CoV-2 sero-epidemiological surveys ^23^, collected essential socio-demographic information. Certified nurses drew a 10 mL blood specimen for serological testing, which was then stored in an icebox before transportation to the Qatar Biobank for long-term storage and subsequent testing. Participants who tested positive for either HBV or HCV infections were connected to additional testing, care, and treatment services at the QRCS.

### Laboratory methods

Serum aliquots were extracted from the preserved specimens at the Qatar Biobank and subsequently transferred to the virology laboratory at Qatar University for serological testing. The sera at both the Qatar Biobank and Qatar University were maintained at −80 °C until employed for serology testing.

#### Current infection with HBV

Current infection with HBV was determined by testing sera for HBsAg using the Mindray CL-900i HBsAg Chemiluminescence Immunoassay Analyzer (CLIA) (Mindray Bio-Medical Electronics, Shenzhen, China). The reported sensitivity and specificity by the Mindray manufacturer are 100% and 99.6% respectively ^24^. The testing results were interpreted in accordance with the manufacturer's guidelines. Sera were categorized as non-reactive if HBsAg concentration was < 0.05 IU/mL and reactive if HBsAg concentration was ≥ 1.00 IU/mL. Intermediate values (0.05 ≤ HBsAg concentration < 1.00 IU/mL) were considered equivocal and underwent centrifugation, followed by duplicate retesting using the same kit. Samples with concentrations < 0.05 IU/mL in both retests were classified as non-reactive, while those with concentrations ≥ 0.05 IU/mL in either of the retests were considered reactive.

Reactive specimens were confirmed using a neutralization test, the Architect HBsAg Qualitative II Confirmatory assay (Abbott Ireland, Sligo, Ireland) ^25,26^. The testing results were interpreted in accordance with the manufacturer's guidelines. The confirmatory result of each specimen was automatically processed by the Architect analyzer, and the outcome was displayed on the analyzer screen.

#### Lifetime infection with HCV

Lifetime infection with HCV was determined by testing sera for HCV antibodies using the Mindray CL-900i HCV Ab CLIA (Mindray Bio-Medical Electronics, Shenzhen, China) ^27^ and also using the Abia HCV Ab Enzyme-linked immunosorbent assay (ELISA) (AB Diagnostic Systems, Berlin, Germany) ^28^. The manufacturers' reported sensitivity and specificity of the Mindray CL-900i HCV CLIA are 99.8% and 99.7%, respectively ^27^, and of the Abia HCV Ab ELISA are 100% and 99.5%, respectively ^28^. We employed two serological assays to assess lifetime HCV infection, aiming to address the limitations of each individual assay. This approach enhanced the overall accuracy, reliability, and robustness of the study results, mitigating the uncertainties associated with each serological test. This also facilitated a comparative analysis of the performance of these two assays.

The testing results were interpreted following the manufacturers' guidelines. Sera tested using the Mindray CL-900i HCV CLIA were categorized as non-reactive if the cut-off index (COI) was < 1.00 and as initially reactive if COI was ≥ 1.00 ^27^. The latter specimens underwent centrifugation, followed by duplicate retesting using the same kit. Specimens with COI < 1.00 in both retests were classified as non-reactive, while those with COI ≥ 1.00 in either of the retests were considered reactive ^27^.

For sera tested with Abia HCV Ab ELISA, an independent cut-off value was calculated for each ELISA 96-well plate per the manufacturer's instructions. Specimens with absorbance/optical density (OD) values below the cut-off were considered negative, while those with OD values equal to or exceeding the cut-off were considered positive. The mean OD value for the negative control was calculated using the equation: mean OD value of the negative control = (OD value B1 + OD value C1 + OD value D1)/3, where B1, C1, and D1 represent the reading of the three negative control wells in each plate. The cut-off was determined using the equation: Cut-off = mean OD value of negative control + 0.24 (0.24 being the constant factor). For assay validation, it was instructed that the OD value of each negative control should be less than 0.2, and the OD value of the positive control should be greater than 2.0, otherwise the testing was to be repeated. In all ELISA runs for this study, the OD value of the positive samples consistently exceeded 0.25 ^28^.

Reactive specimens using Mindray CL-900i HCV CLIA and/or Abia HCV Ab ELISA were confirmed using the Fujirebio INNO-LIA HCV Score line immunoassay (Fujirebio Europe N.V., Gent, Belgium) ^29^. Results were interpreted and classified as positive, negative, or indeterminate using the Fujirebio LiRAS for infectious diseases software ^29^. Indeterminate samples using Fujirebio INNO-LIA HCV Score were all initially dual-tested using Mindray CL-900i HCV CLIA and Abia HCV Ab ELISA. These indeterminate samples were set negative if one of the Mindray CL-900i HCV CLIA or the Abia HCV Ab ELISA test results was non-reactive. Otherwise, indeterminate samples were set positive if both the Mindray CL-900i HCV CLIA and the Abia HCV Ab ELISA test results were reactive.

### Oversight

This study received approval from the Institutional Review Boards of Hamad Medical Corporation, Qatar University, and Weill Cornell Medicine-Qatar. The reporting of the study adhered to the Strengthening the Reporting of Observational Studies in Epidemiology (STROBE) guidelines, as detailed in Table S1 in the Online Supplementary Document.

### Statistical analysis

The study participants were characterized using frequency distributions and measures of central tendency. Probability weights were applied to correct for participants' unequal selection and ensure the sample's representativeness of the broader CMW population. Weights were computed using the population distribution of CMWs by age, nationality, and QRCS center, extracted from the QRCS registered-user database ^16^.

Weights were applied in estimating the prevalence of current infection with HBV and of lifetime infection with HCV. The calculation of the weighted prevalence of current HBV infection involved logistic regression-based multiple imputations with 1,000 iterations. This imputation method incorporated into the analysis three specimens that underwent HBsAg testing and were reactive using the Mindray CL-900i HBsAg CLIA but lacked sufficient sera for confirmatory testing. Histograms were used to illustrate HBsAg concentrations obtained from testing with the Mindray CL-900i HBsAg CLIA and HCV COI values obtained from testing with the Mindray CL-900i HCV CLIA and testing with the Abia HCV Ab ELISA, respectively. The correlation between HCV COI values measured by Mindray CL-900i HCV CLIA and by Abia HCV Ab ELISA was determined using the Pearson correlation coefficient, with the 95% confidence interval (CI) estimated through bootstrapping techniques with 1000 replications.

Associations with current infection with HBV and lifetime infection with HCV were explored through Chi-square tests and univariable logistic regression analyses. Variables with a p-value ≤ 0.2 in the univariable regression analysis were included in the multivariable model. A p-value < 0.05 in the multivariable analysis was considered indicative of a statistically significant association. The study reported unadjusted and adjusted odds ratios (ORs and AORs, respectively), along with their respective 95% CIs and p-values. Interactions were not considered in this analysis.

The comparative diagnostic performance of the Mindray CL-900i HCV CLIA and Abia HCV Ab ELISA in detecting HCV antibodies was assessed by cross-tabulating the results of specimens tested by both assays and calculating positive, negative, and overall percent agreements, along with Cohen's kappa statistic. The kappa statistic quantifies the degree of agreement between any two diagnostic methods beyond what could be expected by chance ^30,31^. All statistical analyses were conducted using Stata/SE version 18.0 (Stata Corporation, College Station, TX, USA).

### Ethics approval

Hamad Medical Corporation, Qatar University, and Weill Cornell Medicine-Qatar Institutional Review Boards approved this retrospective study with a waiver of informed consent.

## Results

### Study population

Table 1 delineates the characteristics of the study participants. Out of the 2641 blood specimens collected during the SARS-CoV-2 antibody prevalence survey ^16^, testing for HBV and HCV was possible for 2528 and 2607 specimens with sufficient sera, respectively.

**Table 1:** Characteristics and prevalence of current infection with HBV and lifetime infection with HCV among study participants.

Characteristics	Current HBV infection				Lifetime HCV infection			
	Tested N (%*)	N^†^	%^‡^ (95% CI^‡^)	Chi-square p-value^£^	Tested N (%*)	N	%^¶^ (95% CI^¶^)	Chi-square p-value
Age (years)								
≤ 29	719 (27.4)	0	0.0 (0.0–0.5)^£^	0.085	745 (27.6)	5	0.8 (0.3–1.8)	0.089
30–39	940 (41.9)	4	0.4 (0.1–1.1)		964 (41.7)	7	0.8 (0.4–1.6)	
40–49	534 (21.6)	4	0.7 (0.2–1.9)		547 (21.5)	3	0.4 (0.1–1.5)	
50–59	249 (7.4)	0	0.0 (0.0–1.5)^£^		260 (7.5)	7	2.3 (1.1–4.8)	
60 +	86 (1.7)	1	0.7 (0.03–6.3)		91 (1.7)	1	0.6 (0.1–4.5)	
Nationality								
All other nationalities**	219 (7.4)	4	1.8 (0.5–4.6)	0.001	230 (7.6)	2	0.7 (0.1–3.5)	< 0.001
Bangladeshi	603 (26.0)	0	0.0 (0.0–0.6)^£^		626 (26.1)	1	0.2 (0.03–1.2)	
Egyptian	86 (3.1)	2	1.9 (0.3–8.1)		90 (3.2)	8	6.5 (3.1–13.1)	
Filipino	99 (2.7)	1	1.2 (0.03–5.5)		101 (2.6)	0	0.0 (0.0–3.6)^£^	
Indian	699 (29.4)	3	0.4 (0.1–1.2)		714 (29.1)	4	0.6 (0.2–1.5)	
Nepalese	552 (21.8)	0	0.0 (0.0–0.7)^£^		565 (21.7)	3	0.6 (0.2–1.9)	
Pakistani	132 (4.9)	0	0.0 (0.0–2.8)^£^		136 (4.9)	4	3.1 (1.1–8.0)	
Sri Lankan	138 (4.7)	0	0.0 (0.0–2.6)^£^		145 (4.8)	1	1.0 (0.1–6.6)	
QRCS center (catchment area within Qatar)								
Fereej Abdel Aziz (Doha-East)	558 (22.1)	1	0.2 (0.00–1.0)	0.914	607 (23.3)	8	1.1 (0.5–2.3)	0.070
Zekreet (North-West)	234 (2.3)	1	0.4 (0.01–2.4)		234 (2.3)	1	0.4 (0.1–3.1)	
Hemaila (South-West; “Industrial Area”)	942 (42.3)	3	0.3 (0.1–0.9)		966 (41.9)	3	0.3 (0.1–1.0)	
Mesaimeer (Doha-South)	794 (33.3)	4	0.5 (0.1–1.3)		800 (32.5)	11	1.2 (0.7–2.2)	
Educational attainment								
Primary or lower	611 (24.8)	0	0.0 (0.0–0.6)^£^	0.001	628 (24.7)	5	0.7 (0.3–1.8)	0.344
Intermediate	416 (17.9)	0	0.0 (0.0–0.9)^£^		429 (17.9)	1	0.3 (0.0–2.0)	
Secondary/high school/vocational	1058 (44.3)	3	0.3 (0.1–0.8)		1087 (44.2)	9	0.8 (0.4–1.5)	
University	348 (12.9)	7	1.9 (0.8–4.1)		365 (13.2)	6	1.5 (0.6–3.5)	
Occupation								
Professional workers^††^	126 (4.6)	1	0.2 (0.02–4.3)	0.768	133 (4.7)	0	0.0 (0.0–2.7)^£^	0.077
Food & beverage workers	85 (3.0)	0	0.0 (0.0–4.2)^£^		91 (3.2)	0	0.0 (0.0–4.0)^£^	
Administration workers	79 (3.0)	1	1.6 (0.03–6.9)		82 (3.0)	1	0.7 (0.1–5.1)	
Retail workers	162 (6.5)	1	0.7 (0.02–3.4)		171 (6.7)	1	0.6 (0.1–4.1)	
Transport workers	410 (16.1)	1	0.1 (0.01–1.4)		429 (16.3)	2	0.2 (0.0–0.9)	
Security workers	57 (2.3)	0	0.0 (0.0–6.3)^£^		60 (2.3)	2	3.2 (0.8–12.2)	
Cleaning workers	102 (4.0)	0	0.0 (0.0–3.6)^£^		104 (4.0)	0	0.0 (0.0–3.5)^£^	
Technical and construction workers^‡‡^	1290 (53.6)	6	0.5 (0.2–1.0)		1313 (52.9)	17	1.2 (0.8–2.0)	
Other workers^§§^	168 (6.8)	0	0.0 (0.0–2.2)^£^		175 (6.9)	0	0.0 (0.0–2.1)^£^	
Total (%, 95% CI)	2528 (100.0)	9	0.4 (0.2–0.7)	–	2607	23	0.8 (0.5–1.2)	–

*CI* confidence interval, *QRCS* Qatar Red Crescent Society.

*Percentage of the sample weighted by age, nationality, and QRCS center. Missing values for socio-demographic variables were excluded from the analysis.

^†^The number of positive individuals was calculated by multiplying the sample size by the proportion of those testing positive in the sample, taking into account probability weights and multiple imputations of three samples with missing values for the confirmatory test.

^‡^The percentage of positive cases within the sample was adjusted by age, nationality, and QRCS center weights and accounted for multiple imputations of three samples with missing values for the confirmatory test. The 95% confidence intervals were calculated using the binomial distribution.

^§^Chi-square test was performed using the original sample before applying multiple imputations.

^¶^Percentage of positive out of the total sample weighted by age, nationality, and QRCS center.

^£^Confidence interval calculated assuming a binomial distribution.

**Includes all other nationalities of craft and manual workers residing in Qatar.

^††^Includes architects, designers, engineers, operation managers, and supervisors among other professions.

^‡‡^Includes carpenters, construction workers, crane operators, electricians, foremen, maintenance/air conditioning/cable technicians, masons, mechanics, painters, pipe-fitters, plumbers, and welders among other professions.

^§§^Includes barbers, firefighters, gardeners, farmers, fishermen, and physical fitness trainers among other professions.

Socio-demographic attributes were similar between CMWs tested for HBV and those tested for HCV (Table 1). Nearly 70% of the study participants were under the age of 40, with a median age of 35.0 years and an interquartile range (IQR) of 29.0–43.0 years. Approximately 40% of participants had educational attainment at intermediate or lower levels, while 44.3% had high school or vocational training education levels. The nationality distribution reflected a predominant presence of Indians (29.4%), Bangladeshis (26.0%), and Nepalese (21.8%), aligning with the broader nationality composition of the CMW population in Qatar ^21^. Over half of the participants (53.6%) were engaged in technical and construction roles, encompassing occupations such as carpenters, crane operators, electricians, masons, mechanics, painters, plumbers, and welders.

### Current infection with HBV

Out of the 2528 specimens tested for HBsAg, 15 were reactive, and 8 were subsequently confirmed positive, while four were confirmed negative. Three samples lacked sufficient sera for HBsAg confirmatory testing but were included in the analysis through logistic regression-based multiple imputations with 1000 iterations (Table 1). The estimated prevalence of current HBV infection among CMWs was 0.4% (95% CI 0.2–0.7%). HBsAg index values in reactive samples ranged from 0.06 to 251.0 (Fig. S1), with a median of 0.33 (IQR 0.07–15.81).

**Figure 1:**
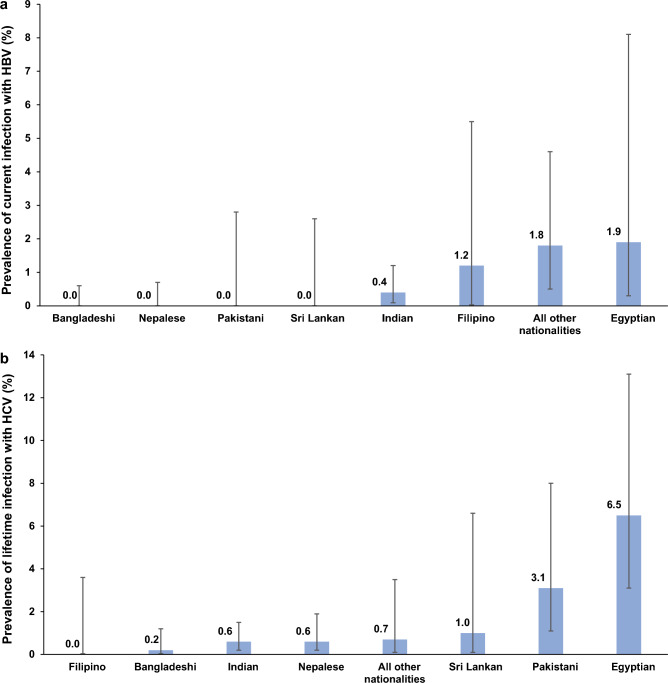
Prevalence of (**a**) current infection with HBV and (**b**) lifetime infection with HCV by nationality group among the craft and manual worker population in Qatar.

Table 1 displays the prevalence of current HBV infection across various socio-demographic characteristics within the population and Fig. 1a shows HBV prevalence by nationality. However, due to the limited number of positive specimens, many socio-demographic strata recorded zero positive cases, hindering a meaningful comparison of prevalence across different strata.

The multivariable regression analysis indicated that educational attainment and occupation were statistically significant factors associated with HBV infection (Table 2). The AOR for CMWs with university-level educational attainment, compared to those with lower educational attainment, was 16.65 (95% CI 3.04–91.05). The AOR was also 11.17 (95% CI 1.53–81.31) for administration and retail workers and 14.20 (95% CI 2.62–77.04) for technical and construction workers, both in comparison to professional and other workers. No other associations reached statistical significance, potentially influenced by the limited number of positive cases.

**Table 2:** Associations with current infection with HBV.

Characteristics	Univariable regression analysis			Multivariable regression analysis	
	OR* (95% CI*)	p-value	F test p-value^†^	AOR* (95% CI*)	p-value^‡^
Age (years)					
≤ 39	1.00		0.185	1.00	
40+	2.59 (0.63–10.55)	0.185		2.71 (0.70–10.58)	0.150
Nationality					
Indian^§^	1.00		0.126	1.00	
Egyptian	4.33 (0.53–35.07)	0.169		1.62 (0.20–13.00)	0.652
Filipino	2.55 (0.36–18.14)	0.351		0.85 (0.10–7.47)	0.881
All other nationalities^¶^	0.47 (0.09–2.58)	0.383		0.89 (0.16–4.92)	0.892
QRCS center (catchment area within Qatar)					
Hemaila (South-West; “Industrial Area”)	1.00		0.881	–	–
Fereej Abdel Aziz (Doha-East)	0.56 (0.06–5.37)	0.612		–	–
Zekreet (North-West)	1.12 (0.12–10.82)	0.923		–	–
Mesaimeer (Doha-South)	1.38 (0.29–6.57)	0.688		–	–
Educational attainment					
Secondary/high school/vocational or lower	1.00		< 0.001	1.00	
University	14.12 (3.25–61.43)	< 0.001		16.65 (3.04–91.05)	0.001
Occupation					
Professional and other workers**	1.00		0.012	1.00	
Administration and retail workers	17.03 (2.36–122.89)	0.005		11.17 (1.53–81.31)	0.017
Technical and construction workers^††^	8.25 (1.58–43.10)	0.012		14.20 (2.62–77.04)	0.002

*AOR* adjusted odds ratio, *CI* confidence interval, *OR* odds ratio, *QRCS* Qatar Red Crescent Society.

*The estimates were adjusted by age, nationality, and QRCS center weights, and accounted for multiple imputations of three samples with missing values for the confirmatory test.

^†^Covariates with p-value ≤ 0.2 in the univariable analysis were included in the multivariable analysis.

^‡^Covariates with p-value < 0.05 in the multivariable analysis were considered to provide statistically significant evidence for an association.

^§^Indian was selected as the reference group because they had low prevalence for current infection with HBV.

^¶^Includes all other nationalities of craft and manual workers residing in Qatar.

**Professional workers include architects, designers, engineers, operation managers, and supervisors among other professions. Other workers include transport workers, security workers, cleaning workers, barbers, firefighters, gardeners, farmers, fishermen, and physical fitness trainers among other professions.

^††^Includes carpenters, construction workers, crane operators, electricians, foremen, maintenance/air conditioning/cable technicians, masons, mechanics, painters, pipe-fitters, plumbers, and welders among other professions.

### Lifetime infection with HCV

Among the 2607 specimens with sufficient sera for HCV testing, 226 (8.7%) underwent exclusive testing with Mindray CL-900i HCV CLIA, 79 (3.0%) exclusively underwent testing with Abia HCV Ab ELISA, and 2302 (88.3%) were tested with both assays. Of the total, 46 specimens were HCV antibody-reactive in either or both tests, with subsequent confirmatory testing identifying 23 specimens as positive. The estimated prevalence of lifetime HCV infection among CMWs was 0.8% (95% CI 0.5–1.2%).

HCV COI values in the 32 antibody-reactive specimens using Mindray CL-900i HCV CLIA ranged from 1.06 to 37.89, with a median of 4.71 (IQR 2.19–17.71; Fig. S2A). HCV COI values in the 37 antibody-reactive specimens using Abia HCV Ab ELISA ranged from 0.26 to 3.84, with a median of 1.54 (IQR 0.33–3.26; Fig. S2B). Among the 23 specimens reactive by both assays, a strong correlation was observed, with a Pearson correlation coefficient of 0.82 (95% CI 0.70–0.93; p-value < 0.001).

Table 1 illustrates the prevalence of lifetime HCV infection across various socio-demographic characteristics within the population, while Fig. 1b depicts HCV prevalence by nationality. Significant variations in prevalence were observed among different nationalities, with the highest recorded among Egyptians at 6.5% (95% CI 3.1–13.1%) and Pakistanis at 3.1% (95% CI 1.1–8.0%). Prevalence also exhibited some variations across age groups, QRCS centers, and occupations.

The multivariable regression analysis identified nationality, QRCS center, and occupation as statistically significant factors associated with lifetime HCV infection (Table 3). The AOR was significantly higher among Egyptians, assessed at 13.41 (95% CI 3.88–46.36), and among Pakistanis, assessed at 5.41 (95% CI 1.20–24.43), compared to Indians. AOR was also significantly higher in a specific geographic location, assessed at 5.03 (95% CI 1.24–20.48) in Fereej Abdel-Aziz compared to Hemaila. Technical and construction workers had an AOR of 6.28 (95% CI 1.76–22.42) compared to professional and other workers. No evidence for an association was found with age or educational attainment.

**Table 3:** Associations with lifetime infection with HCV.

Characteristics	Univariable regression analysis			Multivariable regression analysis	
	OR* (95% CI*)	p-value	F test p-value^†^	AOR* (95% CI*)	p-value^‡^
Age (years)					
≤ 29	1.00		0.101	1.00	
30–39	1.02 (0.32–3.23)	0.975		0.95 (0.27–3.32)	0.941
40–49	0.57 (0.12–2.63)	0.467		0.48 (0.10–2.23)	0.346
50–59	3.14 (0.98–10.05)	0.054		2.59 (0.75–9.01)	0.134
60+	0.86 (0.10–7.43)	0.888		0.59 (0.06–5.77)	0.651
Nationality					
Indian^§^	1.00		< 0.001	1.00	
Bangladeshi	0.30 (0.03–2.72)	0.286		0.29 (0.03–2.63)	0.269
Egyptian	11.93 (3.40–41.86)	< 0.001		13.41 (3.88–46.36)	< 0.001
Nepalese	1.07 (0.24–4.82)	0.930		1.08 (0.22–5.18)	0.924
Pakistani	5.47 (1.33–22.51)	0.019		5.41 (1.20–24.43)	0.028
Sri Lankan	1.70 (0.19–15.42)	0.635		2.51 (0.25–25.74)	0.438
All other nationalities^¶^	0.93 (0.14–6.13)	0.936		1.17 (0.18–7.68)	0.871
QRCS center (catchment area within Qatar)					
Hemaila (South-West; “Industrial Area”)	1.00		0.169	1.00	
Fereej Abdel Aziz (Doha-East)	3.57 (0.92–13.86)	0.066		5.03 (1.24–20.48)	0.024
Zekreet (North-West)	1.39 (0.14–13.46)	0.776		1.41 (0.13–15.26)	0.776
Mesaimeer (Doha-South)	3.86 (1.06–14.05)	0.040		3.39 (0.88–13.07)	0.077
Educational attainment					
Primary or lower	1.00		0.395	–	–
Intermediate	0.39 (0.04–3.43)	0.394		–	–
Secondary/high school/vocational	1.07 (0.34–3.44)	0.903		–	–
University	2.06 (0.57–7.47)	0.271		–	–
Occupation					
Professional and other workers**	1.00		0.049	1.00	
Administration and retail workers	2.24 (0.37–13.68)	0.381		1.78 (0.31–10.43)	0.521
Technical and construction workers^††^	4.30 (1.31–14.13)	0.016		6.28 (1.76–22.42)	0.005

*AOR* adjusted odds ratio, *CI* confidence interval, *OR* odds ratio, *QRCS* Qatar Red Crescent Society.

*Estimates weighted by age, nationality, and QRCS center.

^†^Covariates with p-value ≤ 0.2 in the univariable analysis were included in the multivariable analysis.

^‡^Covariates with p-value < 0.05 in the multivariable analysis were considered to provide statistically significant evidence for an association.

^§^Indian was selected as the reference group because they had low prevalence for lifetime infection with HCV.

^¶^Includes all other nationalities of craft and manual workers residing in Qatar.

**Professional workers include architects, designers, engineers, operation managers, and supervisors among other professions. Other workers include transport workers, security workers, cleaning workers, barbers, firefighters, gardeners, farmers, fishermen, and physical fitness trainers among other professions.

^††^Includes carpenters, construction workers, crane operators, electricians, foremen, maintenance/air conditioning/cable technicians, masons, mechanics, painters, pipe-fitters, plumbers, and welders among other professions.

### Comparison between the Mindray CL-900i HCV CLIA and Abia HCV Ab ELISA assays

Among the 2302 specimens tested with both Mindray CL-900i HCV CLIA and Abia HCV Ab ELISA, 23 were concordant reactive, 2256 were concordant non-reactive, 9 were reactive using Mindray CL-900i HCV CLIA but non-reactive using Abia HCV Ab ELISA, and 14 were non-reactive using Mindray CL-900i HCV CLIA but reactive using Abia HCV Ab ELISA. With Mindray CL-900i HCV CLIA being the comparative assay, the positive, negative, and overall percent agreements were estimated at 71.9% (95% CI 53.3–86.3%), 99.4% (95% CI 99.0–99.7%), and 99.0% (95% CI 98.5–99.5%), respectively. The Cohen's kappa statistic was estimated at 0.66 (95% CI 0.51–0.81), indicating good agreement between the two assays.

## Discussion

Prevalence of current HBV infection among the CMW population stands at only 0.4%, which is lower than the global HBV prevalence of 3.8% ^3,7^. This marked difference mirrors the declining trend observed in South Asian countries, from which many of these workers originate, experiencing a gradual reduction of a few percentage points annually ^32^. The relatively lower HBV prevalence in MENA, compared to other parts of the world ^1,33^, also contributes to this observed low prevalence. This downward trajectory in prevalence may be attributed to the expanding coverage of HBV vaccination during childhood over the past three decades, coupled with widespread enhancements in blood supply screening, injection safety practices, and infection control measures ^32,34^. The low prevalence may also be attributed to the healthy worker effect ^17^ and the health screening of workers before the commencement of work in Qatar.

Prevalence of lifetime HCV infection among CMWs stands at 0.8%, positioning it within the intermediate range observed globally ^35,36^, and comparable to prevalence levels in most countries of MENA ^37–42^. However, this overall prevalence conceals significant variations based on nationality. HCV prevalence was notably high at 6.5% among Egyptians and 3.1% among Pakistanis, aligning with the elevated HCV prevalence documented in Egypt ^13,43–45^ and Pakistan^46–48^, both recognized as having some of the highest levels worldwide ^35,36^. In contrast, the prevalence among other nationalities reflects the comparatively lower prevalence in South Asian countries, the origin of many CMWs ^35,49^.

The study indicated additional noteworthy findings. Specific occupational groups were associated with both HBV and HCV infections, HBV infection showed an association with the level of education, and HCV infection showed an association with geographic location. However, these associations remain unexplained and warrant further investigation. The testing results of both the Mindray CL-900i HCV CLIA and Abia HCV Ab ELISA assays exhibited a close relationship, demonstrating a high level of agreement between the two methods. Intriguingly, the COI and optical density values of both assays were strongly correlated, as evidenced by a Pearson correlation coefficient of 0.82, suggesting consistency in their diagnostic performance.

This study is subject to several limitations. The limited number of specimens positive for HBsAg hindered a detailed analysis of associations with current HBV infection, despite the study's large sample size. Additionally, due to the relatively small number of positive specimens for both HBV and HCV, the 95% CIs for various effects tended to be broad, impacting the precision of the estimates.

While the original study design aimed to employ a probability-based sampling approach for the CMW population, logistical challenges necessitated the adoption of a systematic sampling method focusing on QRCS attendees. To mitigate this deviation, probability-based weights were introduced to produce an estimate reflective of the wider CMW population. To ensure representation of smaller age-nationality strata, a departure from the initial plan—selecting only every fourth attendee—was made, and all individuals in these strata were approached to participate towards the conclusion of the study.

Operational challenges introduced difficulties in tracking and maintaining consistent logs of the response rate by the nurses in the QRCS centers. As a result, an exact estimate of the response rate could not be ascertained; however, it was approximated based on the interviewers' experience to exceed 90%. Although there is a potential that the recruitment scheme may have impacted the generalizability of the study findings, this is considered less likely given the high volume of CMWs frequenting these centers, surpassing 5,000 patients per day ^16^. It is worth noting that these centers function as the primary healthcare providers specifically for CMWs in the country, offering a range of services beyond treating patients, including periodic health certifications, vaccinations, and pre-travel SARS-CoV-2 testing.

## Conclusions

Prevalence of current HBV infection among the CMW population is relatively low, while prevalence of lifetime HCV infection falls within the intermediate range, both in comparison to global and regional levels. However, noteworthy disparities in HCV prevalence are evident based on nationality, particularly with elevated HCV prevalence among Egyptians and Pakistanis. These findings offer insights for the planning of health service provision, the development of policy guidelines, and the implementation of comprehensive HBV and HCV prevention, testing, and treatment programs to curtail HBV and HCV transmission and alleviate the burden of associated diseases. The findings underscore the need to monitor the prevalence of these infections through repeated representative surveys to ensure progress towards achieving the elimination targets by 2030.

### Supplementary Information

Supplementary Information.

## Data Availability

All data are available in aggregate form within the manuscript.

## References

[1] Schweitzer A, Horn J, Mikolajczyk RT, Krause G, Ott JJ (2015). Estimations of worldwide prevalence of chronic hepatitis B virus infection: A systematic review of data published between 1965 and 2013. Lancet.

[2] Stanaway JD (2016). The global burden of viral hepatitis from 1990 to 2013: Findings from the Global Burden of Disease Study 2013. Lancet.

[3] Hsu Y-C, Huang DQ, Nguyen MH (2023). Global burden of hepatitis B virus: Current status, missed opportunities and a call for action. Nat. Rev. Gastroenterol. Hepatol..

[4] Maheshwari A, Ray S, Thuluvath PJ (2008). Acute hepatitis C. Lancet.

[5] Lauer GM, Walker BD (2001). Hepatitis C virus infection. N. Engl. J. Med..

[6] World Health Organization. *Global Health Sector Strategies on, Respectively, HIV, Viral Hepatitis and Sexually Transmitted Infections for the Period 2022–2030*. file:///C:/Users/gm15/Downloads/9789240053779-eng.pdf. Accessed 7 June 2023 (2022).

[7] World Health Organization. *Global Progress Report on HIV, Viral Hepatitis and Sexually Transmitted Infections 2021*. https://www.who.int/publications/i/item/9789240027077. Accessed 4 Dec 2023. (World Health Organization, 2021).

[8] World Health Organization. *Epidemiology of Hepatitis C Virus in the WHO Eastern Mediterranean Region: Implications for Strategic Action*. https://applications.emro.who.int/docs/9789290222866-eng.pdf?ua=1. Accessed 5 Dec 2023 (WHO Regional Office for the Eastern Mediterranean, 2020).

[9] Vermehren J, Park JS, Jacobson IM, Zeuzem S (2018). Challenges and perspectives of direct antivirals for the treatment of hepatitis C virus infection. J. Hepatol..

[10] Horsley-Silva JL, Vargas HE (2017). New therapies for hepatitis C virus infection. Gastroenterol. Hepatol..

[11] El-Akel W (2017). National treatment programme of hepatitis C in Egypt: Hepatitis C virus model of care. J. Viral Hepat..

[12] Flamm SL (2015). Advances in the treatment of hepatitis C virus infection from EASL 2015. Gastroenterol. Hepatol..

[13] Ayoub HH, Abu-Raddad LJ (2017). Impact of treatment on hepatitis C virus transmission and incidence in Egypt: A case for treatment as prevention. J. Viral Hepat..

[14] Chevaliez S (2019). Strategies for the improvement of HCV testing and diagnosis. Expert Rev. Anti Infect. Ther..

[15] Abu-Raddad LJ (2021). Characterizing the Qatar advanced-phase SARS-CoV-2 epidemic. Sci. Rep..

[16] Al-Thani MH (2021). SARS-CoV-2 infection is at Herd immunity in the majority segment of the population of Qatar. Open Forum Infect. Dis..

[17] Al Nuaimi AA (2023). All-cause and COVID-19 mortality in Qatar during the COVID-19 pandemic. BMJ Glob. Health.

[18] Nasrallah GK (2023). Seroprevalence of herpes simplex virus type 1 and type 2 among the migrant workers in Qatar. Virol. J..

[19] Younes N (2023). Seroprevalence of hepatitis E virus (HEV) among male craft and manual workers in Qatar (2020–2021). Heliyon.

[20] Jeremijenko A (2021). Herd immunity against severe acute respiratory syndrome coronavirus 2 infection in 10 communities, Qatar. Emerg. Infect. Dis..

[21] Ministry of Interior-State of Qatar. *Population Distribution by Sex, Age, and Nationality: Results of Kashef Database* (2020).

[22] Planning and Statistics Authority-State of Qatar. *Labor Force Sample Survey*. https://www.psa.gov.qa/en/statistics/Statistical%20Releases/Social/LaborForce/2017/statistical_analysis_labor_force_2017_En.pdf. Accessed 23 May 2022 (2017).

[23] World Health Organization. *Population-Based Age-Stratified Seroepidemiological Investigation Protocol for COVID-19 Virus Infection*. https://apps.who.int/iris/handle/10665/331656. Accessed 15 Apr 2020 (2020).

[24] Mindray. *Mindray HBsAg CL-900i Chemiluminescence Immunoassay Analyzer.**Cat. No. HBsAg112, Shenzhen, China* (2015).

[25] Pasaribu MM (2022). Cutoff value of qualitative HBsAg for confirmatory HBsAg using the chemiluminescence microparticle immunoassay method. Lab. Med..

[26] Abbott. *ARCHITECT HBsAg Qualitative II Confirmatory assay. Abbott Ireland, Sligo, Ireland* (2020).

[27] Mindray. *Mindray CL-900i Chemiluminescence Immunoassay Analyzer.**Cat. No. HCV112, Shenzhen, China* (2015).

[28] AB Diagnostic Systems. *Abia HCV Ab Enzyme-Linked Immunosorbent Assay* (*ELISA*). *Cat. No: DK.067.01.3, Berlin, Germany*. file:///C:/Users/hsc2001/Downloads/DK067013.pdf. Accessed 21 Nov 2023 (2021).

[29] Fujirebio. *Fujirebio INNO-LIA HCV Score Line Immunoassay*. *80538 INNO-LIA HCV Score / 28667 v6 / KEY-CODE: FRI32604, Tokyo, Japan*. https://search.cosmobio.co.jp/cosmo_search_p/search_gate2/docs/IGT_/80538.20170308.pdf. Accessed 21 Nov 2023. (2016).

[30] Fleiss, J. L., Levin, B. & Paik, M. C. *Statistical Methods for Rates and Proportions*. 598–626 (Wiley, 2013).

[31] Aldisi RS (2018). Performance evaluation of four type-specific commercial assays for detection of herpes simplex virus type 1 antibodies in a Middle East and North Africa population. J. Clin. Virol..

[32] Ott JJ, Horn J, Krause G, Mikolajczyk RT (2017). Time trends of chronic HBV infection over prior decades—A global analysis. J. Hepatol..

[33] Ginzberg D, Wong RJ, Gish R (2018). Global HBV burden: Guesstimates and facts. Hepatol. Int..

[34] World Health Organization. *Global Status Report on Blood Safety and Availability 2016*. Report No.: 9789241565431. https://apps.who.int/iris/bitstream/handle/10665/254987/9789241565431-eng.pdf. Accessed 12 Jan 2021 (2021).

[35] Mohd Hanafiah K, Groeger J, Flaxman AD, Wiersma ST (2013). Global epidemiology of hepatitis C virus infection: new estimates of age-specific antibody to HCV seroprevalence. Hepatology.

[36] Lavanchy D (2011). Evolving epidemiology of hepatitis C virus. Clin. Microbiol. Infect..

[37] Chemaitelly H, Mahmud S, Rahmani AM, Abu-Raddad LJ (2015). The epidemiology of hepatitis C virus in Afghanistan: Systematic review and meta-analysis. Int. J. Infect. Dis..

[38] Mohamoud YA, Riome S, Abu-Raddad LJ (2016). Epidemiology of hepatitis C virus in the Arabian Gulf countries: Systematic review and meta-analysis of prevalence. Int. J. Infect. Dis..

[39] Chemaitelly H, Chaabna K, Abu-Raddad LJ (2015). The epidemiology of hepatitis C virus in the fertile crescent: Systematic review and meta-analysis. PLOS ONE.

[40] Fadlalla FA, Mohamoud YA, Mumtaz GR, Abu-Raddad LJ (2015). The Epidemiology of hepatitis C virus in the Maghreb Region: Systematic review and meta-analyses. PloS one.

[41] Chaabna K, Kouyoumjian SP, Abu-Raddad LJ (2016). Hepatitis C virus epidemiology in Djibouti, Somalia, Sudan, and Yemen: Systematic review and meta-analysis. PloS one.

[42] Mahmud S, Akbarzadeh V, Abu-Raddad LJ (2018). The epidemiology of hepatitis C virus in Iran: Systematic review and meta-analyses. Sci. Rep..

[43] Ayoub HH, Chemaitelly H, Kouyoumjian SP, Abu-Raddad LJ (2020). Characterizing the historical role of parenteral antischistosomal therapy in hepatitis C virus transmission in Egypt. Int. J. Epidemiol..

[44] Kouyoumjian SP, Chemaitelly H, Abu-Raddad LJ (2018). Characterizing hepatitis C virus epidemiology in Egypt: Systematic reviews, meta-analyses, and meta-regressions. Sci. Rep..

[45] Mohamoud YA, Mumtaz GR, Riome S, Miller D, Abu-Raddad LJ (2013). The epidemiology of hepatitis C virus in Egypt: A systematic review and data synthesis. BMC Infect. Dis..

[46] Al Kanaani Z, Mahmud S, Kouyoumjian SP, Abu-Raddad LJ (2018). The epidemiology of hepatitis C virus in Pakistan: Systematic review and meta-analyses. R. Soc. Open Sci..

[47] Ayoub HH, Al Kanaani Z, Abu-Raddad LJ (2018). Characterizing the temporal evolution of the hepatitis C virus epidemic in Pakistan. J. Viral Hepat..

[48] Mahmud S, Al Kanaani Z, Abu-Raddad LJ (2019). Characterization of the hepatitis C virus epidemic in Pakistan. BMC Infect. Dis..

[49] Goel A, Rewari BB, Sharma M, Konath NM, Aggarwal R (2022). Seroprevalence and burden of hepatitis C virus infection in WHO South-East Asia Region: A systematic review. J. Gastroenterol. Hepatol..

